# THE IMPACT OF PUBLIC REPORTING ON INEQUITIES IN HIGH-QUALITY HOME HEALTH UTILIZATION

**DOI:** 10.1093/geroni/igac059.1542

**Published:** 2022-12-20

**Authors:** Shekinah Fashaw-Walters, Momotazur Rahman, Gilbert Gee, Vincent Mor, Kali Thomas

**Affiliations:** University of Minnesota School of Public Health, Minneapolis, Minnesota, United States; Brown University, Providence, Rhode Island, United States; UCLA, Los Angeles, California, United States; Brown University, Providence, Rhode Island, United States; Brown University, Providence, Rhode Island, United States

## Abstract

Literature suggests that public reporting of quality may exacerbate disparities in access to high-quality post-acute and long-term care for older adults. The objective of this study was to evaluate the impact of the home health (HH) 5-star ratings on changes in high-quality HH agency (HHA) use, by race, ethnicity, income level, and place-based factors. Using a difference-in-differences framework, we found that after introduction of 5-star ratings adjusted rates of high-quality HHA use increased for all HH users, except for Latinx, Asian American/Pacific Islander, and low-income HH users, and disparities in access to high-quality HHAs were exacerbated for each of these groups. Last, we found that users within predominantly Latinx and lower income neighborhoods had a significantly greater decrease in their use of high-quality HHAs. Policymakers should be aware of the potential unintended consequences of public reporting and should consider adding measures of equity to the publicly reported information.

